# The Merits of Bilateral Application of Middle Ear Implants in Patients With Bilateral Conductive and/or Mixed Hearing Loss

**DOI:** 10.1177/23312165241264466

**Published:** 2024-08-06

**Authors:** Martijn J.H. Agterberg, Louise Straatman, Karl-Ludwig Bruchhage, Tim Jürgens, Daniela Hollfelder, Anke Leichtle

**Affiliations:** 1 Department Otorhinolaryngology, Utrecht Department of Otorhinolaryngology, Head and Neck Surgery, University Medical Center Utrecht, Utrecht, the Netherlands; 2 Department of Otorhinolaryngology, Donders Institute for Brain, Cognition and Behaviour, Radboud University Medical Centre Nijmegen, Nijmegen, the Netherlands; 3 Department of Otorhinolaryngology, Head and Neck Surgery, 54186 University Hospital Schleswig-Holstein, Lübeck, Germany; 4 Department Otorhinolaryngology, University Medical Center (UMC) Utrecht Brain Center, Utrecht, the Netherlands; 5 Institute of Acoustics, University of Applied Sciences Lübeck, Lübeck, Germany

**Keywords:** bilateral conductive hearing loss, binaural hearing, middle ear implant, sound localization

## Abstract

This study investigated sound localization abilities in patients with bilateral conductive and/or mixed hearing loss (BCHL) when listening with either one or two middle ear implants (MEIs). Sound localization was measured by asking patients to point as quickly and accurately as possible with a head-mounted LED in the perceived sound direction. Loudspeakers, positioned around the listener within a range of +73°/−73° in the horizontal plane, were not visible to the patients. Broadband (500 Hz–20 kHz) noise bursts (150 ms), roved over a 20-dB range in 10 dB steps was presented. MEIs stimulate the ipsilateral cochlea only and therefore the localization response was not affected by crosstalk. Sound localization was better with bilateral MEIs compared with the unilateral left and unilateral right conditions. Good sound localization performance was found in the bilaterally aided hearing condition in four patients. In two patients, localization abilities equaled normal hearing performance. Interestingly, in the unaided condition, when both devices were turned off, subjects could still localize the stimuli presented at the highest sound level. Comparison with data of patients implanted bilaterally with bone-conduction devices, demonstrated that localization abilities with MEIs were superior. The measurements demonstrate that patients with BCHL, using remnant binaural cues in the unaided condition, are able to process binaural cues when listening with bilateral MEIs. We conclude that implantation with two MEIs, each stimulating only the ipsilateral cochlea, without crosstalk to the contralateral cochlea, can result in good sound localization abilities, and that this topic needs further investigation.

## Introduction

An established treatment for patients with bilateral conductive hearing loss (BCHL) is the provision of one or two bone-conduction devices (BCDs). Most patients with BCHL benefit from bilateral implantation in comparison to the unilateral provision of BCDs. Studies in adult patients (Bosman et al., 2001; Caspers et al., 2022; Priwin et al., 2004), and in children (Den Besten et al., 2020; Dun et al., 2013) demonstrated improvements in sound localization abilities and speech intelligibility with bilateral BCDs relative to a unilateral BCD. However, bilateral application of BCDs does only result in lateralization of sound stimuli (Shiraishi, 2021), that is, the general ability to judge from which hemisphere the sound is coming from, and not precise localization. Whether bilateral application of BCDs can restore binaural hearing in the sense that both interaural time differences (ITDs) and interaural level differences (ILDs) as in normal-hearing are exploited, remains an open question (Agterberg, Hol, et al., 2011).

A lack of proper integration of ITDs and ILDs in patients with BCHL may be due to several factors. One potentially important factor is the stimulation of the contralateral ear due to the limited transcranial attenuation of bone-conducted sounds (Stenfelt, 2012). This was demonstrated directly in human cadaveric specimen (Farrell et al., 2017). Consequently, a single BCD stimulates both the contralateral and ipsilateral cochlea simultaneously, leading to contamination of ILD/ITD processing.

In contrast, a middle ear implant (MEI) stimulates only the respective ipsilateral cochlea due to the attachment of the transducer to the middle ear ossicle chain or round window. Potentially bilateral application of MEIs may result in optimal sound localization with precision in azimuth of a few degrees (Blauert, 1997; Middlebrooks & Green, 1991), because transcranial crosstalk, as present for BCDs is virtually absent for bilateral MEIs.

MEIs were originally developed for treating pure sensorineural hearing loss (Snik & Cremers, 1999). However, the indication criteria for MEIs such as the vibrant soundbridge (VSB) have been extended toward patients with mixed hearing losses consisting of moderate BCHL (Dumon et al., 2009), and to patients with pure conductive hearing loss (Frenzel, 2018). However, due to several reasons bilateral implantation of MEIs is scarce. In most countries MEIs are not provided at all due to the relatively high costs for implantation and aftercare. In the United States and in the Netherlands MEIs are not reimbursed and are only provided as last resort solution. Therefore, in the Netherlands, MEIs are only provided unilaterally. In other countries like Germany or Poland MEI systems are provided more regularly. Still bilateral implantation in these countries is rare, and bilateral MEIs are only provided when both ears meet the implantation criteria and other options, such as conventional hearing aids do not result in sufficient benefit. Consequently, the number of persons implanted with two MEIs is very small, even in clinics that implant MEIs regularly.

The aim of the present study is to investigate whether or not patients with bilateral BCHL who received bilateral MEIs, demonstrate appropriate processing of binaural cues, by testing whether localization performance can be as good as sound localization abilities of normal-hearing subjects.

## Materials and Methods

### Patients With BCHL

Six patients (two female and four male) diagnosed with BCHL and bilaterally implanted with an MEI (Vibrant Soundbridge^®^, MED-EL) participated in the present study. The inclusion criterion was a word recognition score (WRS) ≥ 60% in the Freiburg monosyllables test (Hahlbrock, 1953) in quiet. The average age was 40 years (SD 20 years) and aided hearing experience varied from 2 to 156 months. All patients used the Samba 2 Hi processors, which were individually fine-tuned, based on the patient's comments (according to Bruchhage et al., 2023). Patient characteristics are presented in Table 1.

**Table 1.** Patient and Audiological Characteristics

ID	Sex	Implant	FMT Coupling	Audioprocessor	Study participation age (years)	Age at implantation (years)	Time after activation (months)	Etiology	Previous operations	4PTA			AC aided SF (%) monaural			AC aided SF (%) bilateral		
										BC	AC	SF	65 dB	80dB	65/65 dB	65 dB	80 dB	65/65 dB
P1_R	male	VORP 503	Sp	Samba 2 Hi	63	60	36	Stenosis	WEC, twice 2007	20.0	45.0	31.3	95	100	55	85%	90%	75%
P1_L		VORP 503	Sp	Samba 2 Hi		62	9	Stenosis	WEC, 2021	18.8	61.3	30.0	90	100	55			
P2_R	Male	VORP 503	St	Samba 2 Hi	18	16	24	COME	Tymp	18.8	61.3	25.0	95	100	55	85%	90%	75%
P2_L		VORP 503	Sp	Samba 2 Hi		16	27	COME	Tymp	22.5	51.3	32.5	90	100	55			
P3_R	Male	VORP 503	RW	Samba 2 Hi	44	44	2	MHL	None	32.5	58.8	28.8	95	100	60	95%	100%	85%
P3_L		VORP 503	RW	Samba 2 Hi		44	5	MHL	None	27.5	53.8	21.3	80	100	70			
P4_R	Male	VORP 503	SH	Samba 2 Hi	63	40	156	SNHL	none	56.3	58.8	37.5	50	80	40	55%	60%	10%
P4_L		VORP 503	LP	Samba 2 Hi		62	2	SNHL	none	53.8	78.8	47.5	60	75	10			
P5_R	Female	VORP 502	St	Samba 2 Hi	12	1.5	120	CAA	Ear reconstruction 2020	17.5	78.8	13.8	85	100	55	90%	95%	65%
P5_L		VORP 502	St	Samba 2 Hi		1.5	120	CAA	Ear reconstruction 2020	13.8	73.8	10.0	95	95	70			
P6_R	Female	VORP 503	RW	Samba 2 Hi	40	34	82	COME	Tymp 2010; 2015 with RC	36.3	88.8	38.8	95	95	85	95%	100%	70%
P6_L		VORP 503	RW	Samba 2 Hi		34	84	COME	Tymp 2010; 2015 with RC	35.0	67.5	31.3	75	100	65			

*Note*. R = right ear, L = left ear, St = Stapes, SP = short process of incus, LP = long process of incus, SH = stapes head, RW = round window, SNHL = sensorineural hearing loss, CAA = congenital aural atresia, COME = chronic otitis media epitympanalis, MHL = mixed hearing loss, TYMP = tympanoplasty, RC = radical cavity, SF = sound field, 4PTA = average of 0.5, 1, 2, 4 kHz, BC = bone conduction, AC = air conduction; FMT = floating mass transducer.

### Listening Conditions

Patients with BCHL were tested in four listening conditions; (a) unilateral aided with MEI left, (b) unilateral aided with MEI right, (c) bilaterally aided with MEI, (d) unaided. Listening conditions 1, 2 and 3 were randomized, the unaided condition was always measured last.

### Audiometric Tests

Following the clinical routine measurements pure tone averages of bone conduction (4PTA_BC_), air conduction (4PTA_AC_), and sound field thresholds (4PTA_SF_) were performed with a clinical audiometer (Auritec AT 1000, Hamburg, Germany) in a sound-attenuated chamber according to ISO 8253-1 (ISO 8253-1, 2010). The pure-tone audiometry and speech audiometry were performed with calibrated headphones (Beyerdynamic DT48A; Berlin, Germany) at frequencies of 0.5, 1, 2, and 4 kHz in aided monaural (plugging and masking the contralateral ear) and binaural condition. WRS was determined by using the Freiburger monosyllables test in quiet at sound pressure levels of 65 dB and 80 dB and in broadband noise at 65 dB speech level.

### Sound Localization Setup and Stimuli

Sound localization tests were performed in a sound-isolated anechoic mobile auditory lab (Wasmann et al., 2020). Listeners were positioned in a chair at 1.2 m from 24 speakers (Genelec 8010, Iisalmi, Finland) positioned around the listener within a range of +40°/−30° in the vertical plane and ±73° in the horizontal plane, which were invisibly hidden behind an acoustically transparent fabric. An LED at 0° azimuth and 0° elevation served as the central fixation point. Head movements toward the perceived sound location were recorded via infrared cameras (Smarttrack, ART, Munich, Germany) tracking a head-mounted frame with six reflectors. Sound stimuli consisted of broadband (0.5–20 kHz) Gaussian noise bursts. All stimuli had a stimulus duration of 150 ms with 10 ms on-/offset ramping. A complete trial consisted of 45 stimuli (15 stimuli per sound level) and sound levels (45, 55 and 65 dB, A-weighted) were presented interleaved in randomized order to minimize the use of monaural level cues.

The mobile laboratory was placed close to the entrance of the hospital. The listener was positioned with the central loudspeaker at elevation 0°, azimuth 0°, at the height of the listener's ears, by adjusting the height of the chair. The setup was calibrated and checked immediately before every new series of measurements. Head saccades were stored and monitored online by the experimenter. When the first head-saccade did not start between −5 and 5° azimuth an adjustment of the markers on the classes was made and the head-saccade was checked again. All patients signed informed consent to participate in this study. The present study was performed in accordance with the Declaration of Helsinki and approved by the ethics committee of the University Clinics Schleswig-Holstein (AZ20-019).

### Patient Instructions

To keep the head pointing straight ahead during stimulus presentation, the listeners were instructed to point with a head-mounted green LED to the red LED in front of the subject, and then make a head movement toward the perceived sound location as fast and as accurately as possible. Visual orientation about their momentary head direction was provided using the head-mounted LED which was visible always in the nose direction of the subject. The subject was then asked to hold the perceived sound direction for at least 2 s. More details about this sound localization method can be found in Vogt et al. (2018) and Wasmann et al. (2020). To familiarize the patient with the setup, five stimuli were presented and it was checked whether all information was processed. Oral feedback was provided when the task was not performed optimally. For example, subjects should respond immediately with a head-saccade after stimulus presentation. During the test the patients pressed a button to initiate the presentation of the next sound. They were asked to respond with a head-movement toward the perceived sound direction, as fast and as accurately as possible. This method has been shown to be accurate for subjects across the entire life span (Otte et al., 2013).

### Comparison With Patients With Bilateral BCDs

For reference purposes, the data of the patients participating in an earlier study (Den Besten et al., 2020) are presented. These patients were children provided bilaterally with percutaneous BCDs at least one year before the conduction of the study, who performed the same localization task.

### Data Analysis

Velocity and the final head position were used to determine the head-saccade response (Agterberg et al., 2019; Agterberg, Snik, et al., 2011; Den Besten et al., 2020). Data were analyzed for each patient and each condition separately and final stable head-saccade responses are shown as individual target-response plots. In case of perfect localization, the data points will demonstrate a diagonal orientation of data points and the mean absolute error (MAE) is below 10°, equaling the performance of normal hearing subjects (Vogt et al., 2018).

MAE and *SD* values were calculated, and for statistical analyses paired *t*-tests and independent samples *t*-tests were performed.

## Results

### Localization Instead of Lateralization When Listening With Two MEIs

**Figure 1.**
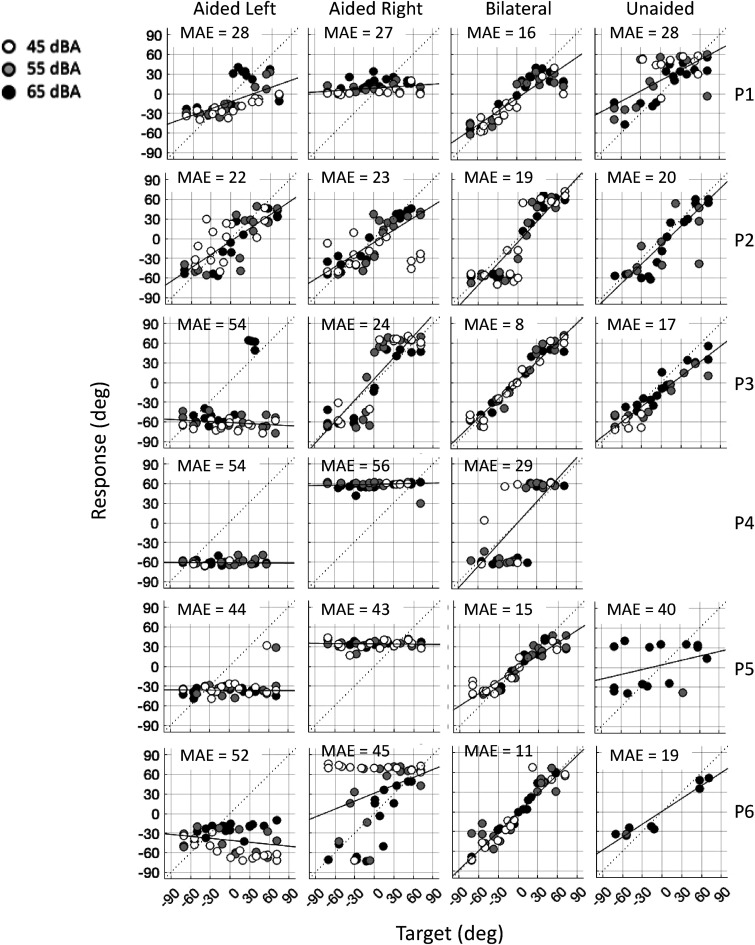
Target-response plots for all patients (rows) in every condition (columns). The left-side column shows the unilateral aided left condition, the middle columns the unilateral aided right and bilateral aided conditions, and the ride-side column the unaided condition. Each data point represents a localization response to a target (white: 45 dBA, gray: 55 dBA, black: 65 dBA). Best-fit linear regression and MAE are plotted in each subfigure.

Figure 1 shows the individual target-response plots for all patients in the unilateral left aided, unilateral right aided, bilaterally aided, and unaided condition. Each data point represents the endpoint of a head-movement toward the corresponding stimulus. The sound levels are indicated by different colors and symbols. Black is 65 dB(A), gray 55 dB(A) and open symbols represent the stimuli presented at 45 dB(A). The best linear fit between target and response is indicated by the black regression line. MAEs in degrees are indicated within each subpanel. The figure depicts poor performance in sound localization in the unilateral conditions for all patients. In the bilateral condition endpoints of head-movements close to the dotted diagonal line were seen in patients P1, P3, P5, and P6, indicating accurate sound localization. Patients P2 and P4 are demonstrating a bimodal distribution of the data, that is, endpoints of head-movements fall either to the far-left or far-right (classified as lateralization rather than localization of sounds).

MAE = mean absolute error.

A typical example of a patient who is perceiving all stimuli in the unilateral conditions at one particular location is patient P5. This patient perceived all stimuli in the unilateral aided left condition, at the left side (bias = −36°, MAE = 44°), and in the unilateral aided right condition at the right side (bias = 34°, MAE = 43°). The bias for the different sound levels was similar in both unilateral conditions. In the bilaterally aided condition, the bias decreased to 2° and the MAE decreased to 15°.

Patient P6 perceived all stimuli in the unilateral aided left condition also at the left side (bias = −41°, MAE = 52°). However, in the unilateral aided right condition, most but not all stimuli, were perceived at the right side (bias = 31°, MAE = 45°). P6 demonstrates a clear level-dependent bias in the unilateral conditions: High levels (black dots) were more centrally perceived than low levels (white dots). In the bilaterally aided condition, the bias decreased to 2° and the MAE decreased to 10.5°. Patient P6 did only perceive nine of the 45 presented stimuli (at the highest sound level) when both VSBs were turned off (unaided condition).

### Comparison Bilateral MEIs With Bilateral BCDs

**Figure 2.**
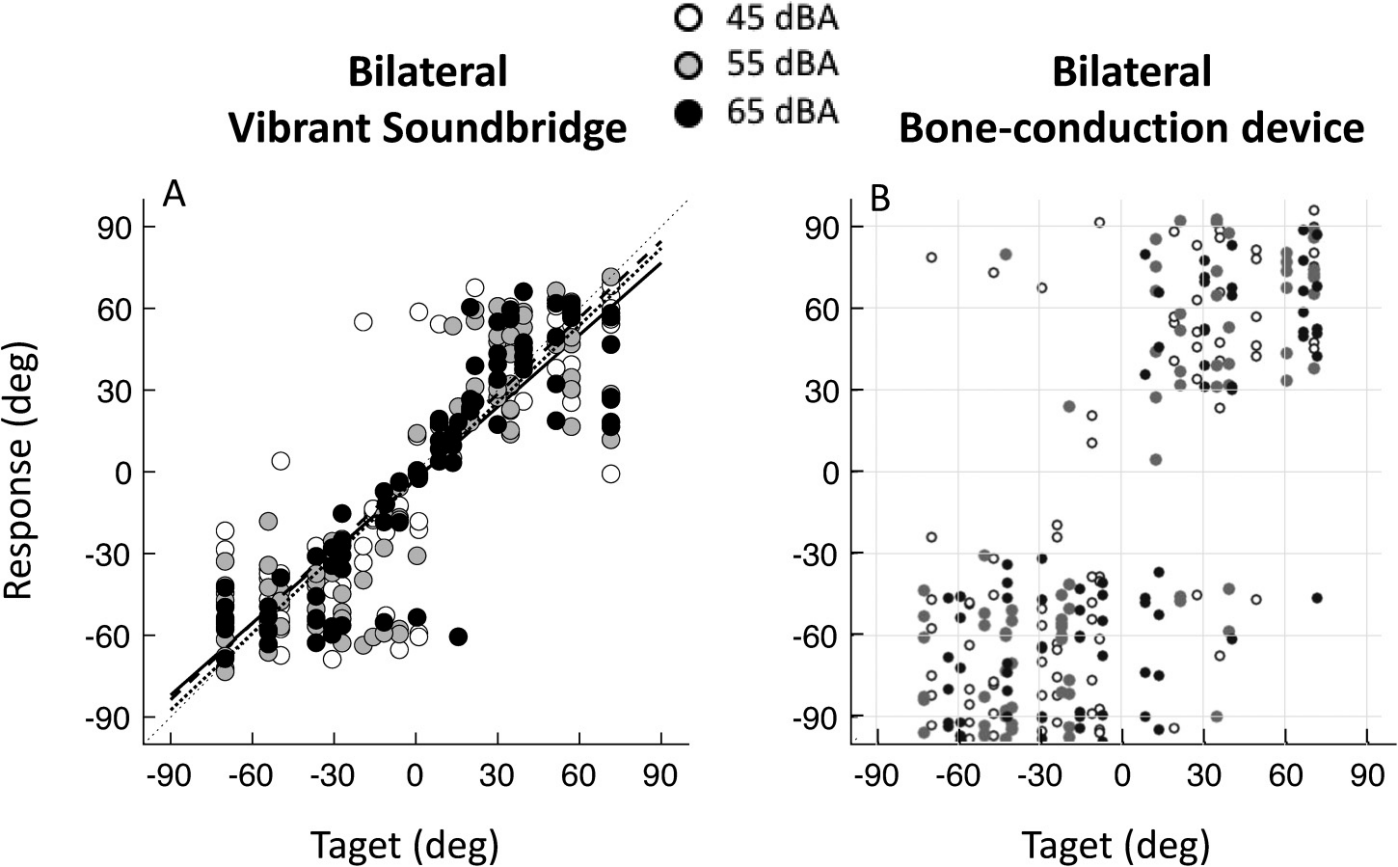
Pooled azimuth target-response plots for all patients with bilateral MEIs compared to BCHL patients implanted with bilateral BCDs (adapted from Den Besten et al., 2020). Each data point represents a localization response to a target (white: 45 dBA, gray: 55 dBA, black: 65 dBA). Best-fit linear regression lines are indicated per level (dotted line for 45 dBA, gray line for 55 dBA, and black line for 65 dBA) for the bilateral MEI data.

Figure 2 shows the pooled target-response data for all subjects compared to previously published data about sound localization with bilateral BCDs (Den Besten et al., 2020). In case of perfect sound localization all data point would fall on the diagonal line. The comparison indicates the striking difference between patients implanted with bilateral MEIs and patients implanted with bilateral BCDs. For the highest sound levels (black symbols) there is a clear diagonal orientation of the data points within the patients with bilateral MEIs, but not with bilateral BCDs.

MEI = middle ear implant; BCHL = bilateral conductive hearing loss; BCD = bone-conduction device.

In Table 2, the MAE scores of all MEI patients, of the patients with congenital BCHL from the previous study performed by Den Besten et al. (2020), and of seven normal hearing subjects from Vogt et al. (2018), are listed. The individually inferred MAEs were found to be normally distributed for each subject group and each condition using the Shapiro–Wilk test. Patients implanted with bilateral MEIs have a statistically significant smaller MAE (*M* = 16°, *SD* = 7°) than patients with bilateral BCDs (*M* MAE = 37°, *SD* = 11°, *t*-test, *p* < .005). The MAE for patients with bilateral MEIs is close to the range of normal-hearing listeners (*M* = 9°, *SD* = 3°).

**Table 2.** The mean absolute errors (MAEs) for all patients in the unilateral left, unilateral right, bilateral and unaided condition. For comparison the MAEs of previous published data (Den Besten et al., 2020), and normal hearing data (Vogt et al., 2018) are presented.

	Anke			
	Aided left	Aided right	Bilateral	Unaided
P1 MEIs	27.7	36.5	15.8	28.1
P2 MEIs	22.4	22.8	18.8	19.8
P3 MEIs	54.4	23.9	7.8	16.8
P4 MEIs	53.5	55.8	29	X
P5 MEIs	44	43.1	14.7	39.7
P6 MEIs	52.1	45	10.5	19.1
*M*	42.4	37.9	16.1	24.7
*SD*	14.0	12.8	7.4	9.4
P1 BCDs	66.5	59.4	38.4	
P3 BCDs	70.6	66.8	42.7	
P6 BCDs	51.6	55	18.6	
P7 BCDS	57.6	X	52.9	
P8 BCDs	71.7	82.7	27.9	
P9 BCDs	51.3	54.9	36.3	
P10 BCDs	45.9	67.3	44.7	
*M*	59.3	64.4	37.4	
*SD*	10.3	10.5	11.3	
	Normal hearing			
NH1	14.6			
NH2	7.3			
NH3	5.2			
NH4	8.8			
NH5	5.9			
NH6	8.3			
NH7	10.9			
*M*	8.7			
*SD*	3.2			

*Note.* MEI = middle ear implant; BCD = bone-conduction device.

### Unilateral Aided

**Figure 3.**
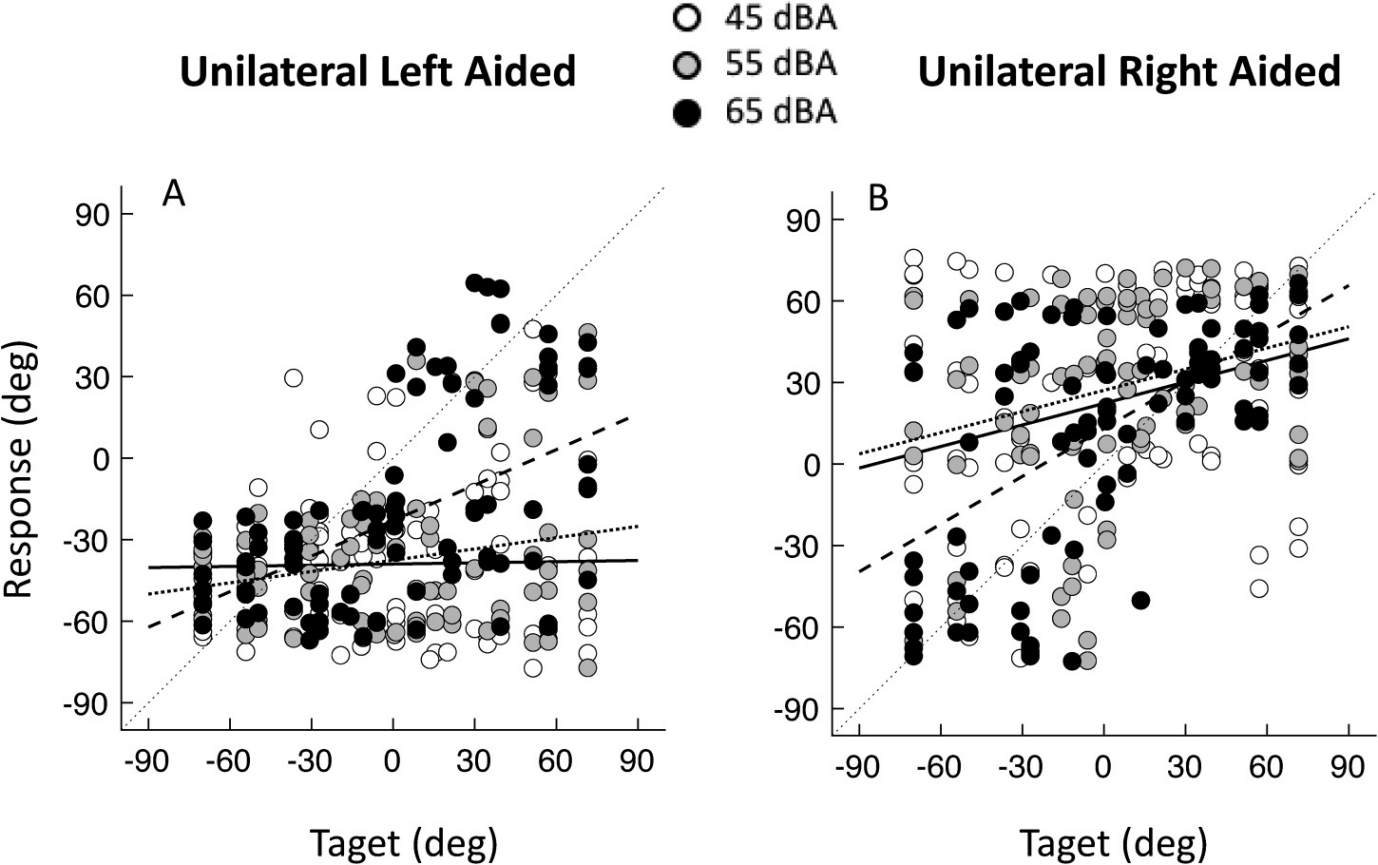
Pooled azimuth target-response plots for all patients in the aided left and aided right conditions.

Typically, patients perceive all sounds coming from only one side when one of their hearing implants is turned off (Denanto et al., 2022; Den Besten et al., 2020). Figure 3 shows that when one MEI is turned off, stimuli are not perceived as coming from the side of the active MEI only, but there are many contralateral responses, especially at high sound levels of 55 dB(A) or 65 dB(A). However, overall performance in these unilateral conditions is poor compared to the bilaterally aided condition with MEIs. The MAE in the unilateral aided left (*M* = 42°, *SD* = 14°) and in the unilateral aided right condition (*M* = 40°, *SD* = 13°) are significantly poorer than in the bilateral MEI condition (*M* = 16°, *SD* = 7°, paired *t*-test *p* < .005).

### Unaided Sound Localization Abilities

**Figure 4.**
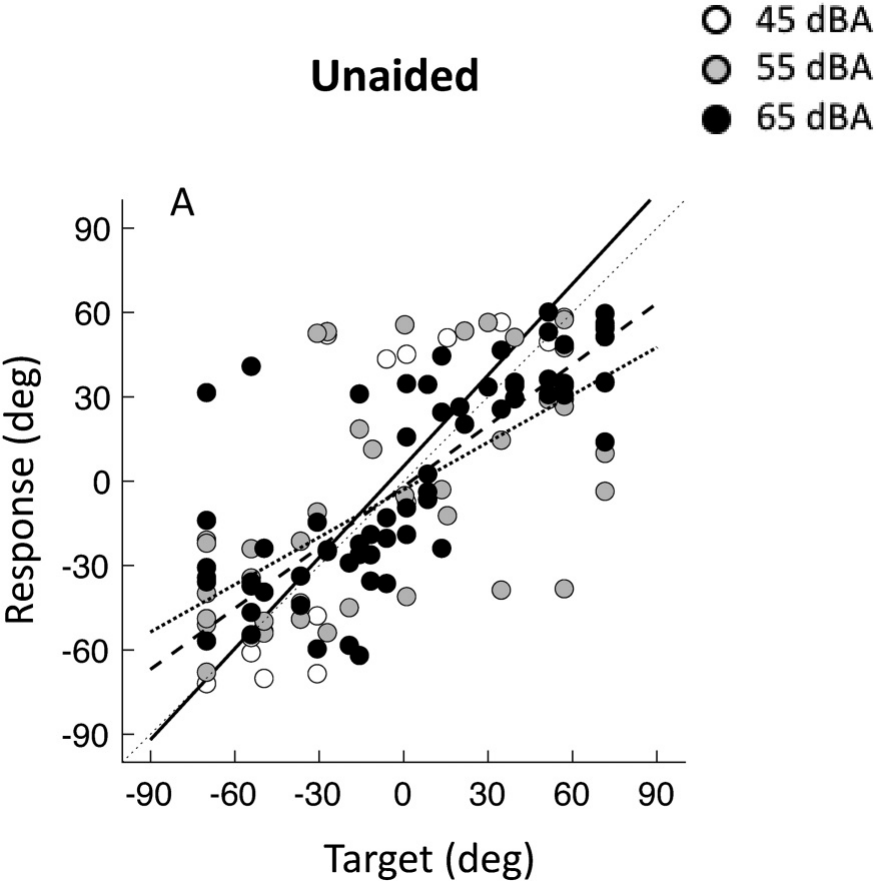
Pooled azimuth target-response plot of all patients in the unaided condition. Note that P4 is not included because this patient did not perceive any of the stimuli in the unaided condition. Each data point represents a localization response to a target (white: 45 dBA, gray: 55 dBA, black: 65 dBA). Best-fit linear regression lines are indicated per level (dotted line for 45 dBA, gray line for 55 dBA and black line for 65 dBA).

Figure 4 shows target-response plots of all patients bilaterally implanted with MEIs in the unaided condition, that is, with their sound processors turned off. A significant number of sound stimuli was perceived by most patients in the unaided condition with fairly good accuracy. The MAE in the unaided condition were 24° for 45 dB(A), 30° dB for 55 dB(A) and 21° for 65 dB (A). Patients P1 and P3 (right-most column in Figure 1) did perceive even some stimuli presented at the lowest sound level (45 dBA). These MAEs, however, are substantially higher than the MAEs in the bilaterally aided condition (Figure 1).

## Discussion

The present study demonstrates that patients with BCHL who are bilaterally implanted with MEIs can show good azimuth sound-localization behavior. Five out of six patients improved with respect to the unaided or single-aided condition indicated as substantially lower MAEs. Four out of five patients showed MAEs close to normal-hearing performance (MAEs around 10°, Wasmann et al., 2020) for all sound levels presented here.

Patient P4, who was tested 2 months after implantation of the second ear, demonstrated hemispheric lateralization of sounds only, while patient P3, who also received the second implant 2 months before testing sound localization, demonstrated almost perfect localization abilities (Figure 1). Patient P4 has been listening with one MEI for 13 years while patient P3 was implanted 2 months after activation of the first implant (see Table 1). Therefore, the long period of monaural input for P4 could have contributed to this patient's poor localization performance in the bilateral aided condition.

VSBs stimulate the respective structure of the middle ear where the floating mass transducer (FMT) is coupled acoustically, such that the stimulation is directly transferred into the inner ear (Frenzel et al., 2015; Vogt et al., 2018). In comparison to bilateral BCDs, transcranial crosstalk is therefore virtually absent. This may lead to a more faithful representation of binaural cues that can be exploited for sound localization in bilateral MEI users in comparison to unaided patients, or persons with bilateral BCDs. ILDs will probably be better represented due to appropriate level amplification in both MEIs without cross-talk, and ITDs will either be better represented due to more salient (amplified) landmarks in the presented sounds that can be used for ITD extraction within the envelopes or ITD fine-structure processing in the brainstem enabled by matching processing latencies in the two devices. Stimulation of the contralateral cochlea by cross-talk is mentioned as a possible limitation in localization with BCDs or MEIs for patients with unilateral conductive hearing loss (Vogt et al., 2018), but to our knowledge, not for patients with BCHL. With respect to the possible exploitation of ITDs, the processing latency of the two MEIs is important and needs further investigation. The exact processing latency of each MEI is not known to the authors.

In patients P1, P3, P5, and P6 the two MEIs (right and left) are coupled to the same middle-ear structure. No systematic ITD bias is expected for these patients. However, P2 has different FMT-couplings right and left, and P2 also shows a lateralization pattern rather than an accurate localization pattern of results with raised MAEs. Hence, it may be possible that different couplings of MEIs across ears create latency biases that do not allow the patient to utilize ITD cues for sound localization. The inability to use ITD cues may then lead to poorer sound localization which is a phenomenon also known for example in bilateral cochlear implant patients (Williges et al., 2018).

The two MEI sound processors in each patient in the present study are not technically synchronized with high temporal precision. An equalization of possible latency differences across ears in patients with couplings to different middle-ear structures across ears, therefore, may lead to improved sound localization, as has recently been shown for patients with cochlear implants and contralateral hearing aids (Zirn et al., 2019) and patients with different hearing aid processing types across ears (Keidser et al., 2006). However, in the study performed by Zirn et al. (2019) loudspeakers were positioned 30 degrees apart, and therefore monaural level cues, monaural spectral cues and/or the use of the direction-specific timbre of sound, might have dominated the localization behavior in these conditions (Arras et al., 2022).

Unexpectedly good localization performance was found for the patients with bilateral MEIs in the unaided condition (i.e., when their sound processors were turned off) using high (65 dB and for some 55 dB) sound level presentation. This indicates that a nonnegligible portion of sound reaches the inner ear of MEI users via air conduction even when their implants are switched off. When a MEI implant is turned off residual hearing is affected at most by just a couple of dB (Tringali et al., 2011) meaning that the device doesn’t have to be surgically removed when it fails to work properly.

Patients P1 and P2 had better hearing at the low than at the high frequency and therefore accurate ITD processing may have contributed to the accurate localization abilities of these two patients (Blauert, 1997). ILD cues, which are most prominent at high frequencies, may be less pronounced in all patients when listening without their hearing devices due to missing amplification at the high frequencies. In turn, usage of bilateral MEIs may therefore give persons with BCHL the ability to reliably exploit also ILD cues in addition to ITD cues for more accurate sound localization.

The clinical relevance of accurate sound localization abilities in the everyday life of patients has still to be explored. The ultimate goal of bilateral implantation is the binaural processing of sounds, which results in optimal sound localization and improved speech understanding of noise. Whether patients with bilateral MEIs indeed experience also better speech understanding in noisy environments needs further investigation (Dutt et al., 2002; Weiss et al., 2016).

In comparison to normal-hearing listeners, monaural cues provided by sound reflections in the pinna, head, and torso are absent for MEI users due to the position of the microphone in the behind-the-ear speech processor case. These cues are of less importance for azimuthal sound localization (Arras et al., 2022; Stevenson-Hoare et al., 2022; Van den Bogaert et al., 2011).

There are a few limitations in the comparison of the patients with bilateral MEIs here and the patients with bilateral BCDs in Den Besten et al. (2020). First, the bone-conduction audiometry thresholds of the patients in Den Besten et al. (2020) are normal and those of the patients in the present study show mild to moderate sensorineural hearing impairment (17.5–36.3 dB HL) in agreement with the indication criteria of the VSB. Since mild-to-moderate hearing impairment does not affect azimuthal sound localization (Otte et al., 2013), this difference is unlikely to affect sound localization here. Furthermore, the patients in Den Besten et al. (2020) were children, whereas the present study tested adults (except for P5). While sound localization ability generally improves through early childhood (Van Deun et al., 2009), individual ability surely depends on exposure to interaural cues and training in exploiting them guided by visual feedback. Therefore, we cannot fully rule out that localization ability might improve in the patients of Den Besten et al. (2020) during adolescence. However, the fact that the patients of Den Besten et al. (2020) had at least 1 year (and an average of 5 years) of experience with bilateral BCDs in their everyday lives makes a substantial improvement in sound localization through more years of experience less likely.

This is the first study demonstrating almost accurate sound localization abilities, similar to normal-hearing listeners, when listening with bilaterally applied active transcutaneous MEIs, indicating that processing of binaural cues is feasible with bilateral VSBs.
